# Regulation of dendritic spines in the amygdala following sleep deprivation

**DOI:** 10.3389/frsle.2023.1145203

**Published:** 2023-04-03

**Authors:** Lindsay Rexrode, Matthew Tennin, Jobin Babu, Caleb Young, Ratna Bollavarapu, Lamiorkor Ameley Lawson, Jake Valeri, Harry Pantazopoulos, Barbara Gisabella

**Affiliations:** 1 Department of Psychiatry and Human Behavior, University of Mississippi Medical Center, Jackson, MS, United States,; 2 Program in Neuroscience, University of Mississippi Medical Center, Jackson, MS, United States

**Keywords:** amygdala, sleep, memory consolidation, growth hormone, dendritic spines

## Abstract

The amygdala is a hub of emotional circuits involved in the regulation of cognitive and emotional behaviors and its critically involved in emotional reactivity, stress regulation, and fear memory. Growing evidence suggests that the amygdala plays a key role in the consolidation of emotional memories during sleep. Neuroimaging studies demonstrated that the amygdala is selectively and highly activated during rapid eye movement sleep (REM) and sleep deprivation induces emotional instability and dysregulation of the emotional learning process. Regulation of dendritic spines during sleep represents a morphological correlate of memory consolidation. Several studies indicate that dendritic spines are remodeled during sleep, with evidence for broad synaptic downscaling and selective synaptic upscaling in several cortical areas and the hippocampus. Currently, there is a lack of information regarding the regulation of dendritic spines in the amygdala during sleep. In the present work, we investigated the effect of 5 h of sleep deprivation on dendritic spines in the mouse amygdala. Our data demonstrate that sleep deprivation results in differential dendritic spine changes depending on both the amygdala subregions and the morphological subtypes of dendritic spines. We observed decreased density of mushroom spines in the basolateral amygdala of sleep deprived mice, together with increased neck length and decreased surface area and volume. In contrast, we observed greater densities of stubby spines in sleep deprived mice in the central amygdala, indicating that downscaling selectively occurs in this spine type. Greater neck diameters for thin spines in the lateral and basolateral nuclei of sleep deprived mice, and decreases in surface area and volume for mushroom spines in the basolateral amygdala compared to increases in the cental amygdala provide further support for spine type-selective synaptic downscaling in these areas during sleep. Our findings suggest that sleep promotes synaptic upscaling of mushroom spines in the basolateral amygdala, and downscaling of selective spine types in the lateral and central amygdala. In addition, we observed decreased density of phosphorylated cofilin immunoreactive and growth hormone immunoreactive cells in the amygdala of sleep deprived mice, providing further support for upscaling of dendritic spines during sleep. Overall, our findings point to region-and spine type-specific changes in dendritic spines during sleep in the amygdala, which may contribute to consolidation of emotional memories during sleep.

## Introduction

Sleep is critical for the regulation of emotional memory consolidation, and disturbances in sleep and memory processing along with alterations in dendric spine numbers are widely reported in people with psychiatric disorders (Ford and Kamerow, 1989; Krakow et al., 2000; Ohayon and Shapiro, 2000; Krystal et al., 2008, 2016; Dorph-Petersen et al., 2009; Sweet et al., 2009; Murray and Harvey, 2010; Gruber et al., 2011; Penzes et al., 2011; Duman and Aghajanian, 2012; Ebdlahad et al., 2013; Germain, 2013; Glausier and Lewis, 2013; Licznerski and Duman, 2013; Shelton et al., 2015; Koffel et al., 2016; Dolsen et al., 2017; MacDonald et al., 2017; Soehner et al., 2017; Forrest et al., 2018; Gisabella et al., 2021). Sleep is critically involved in strengthening memories (for review see Stickgold, 2005). However, the morphological and molecular processes involved in memory consolidation during sleep are still not well understood. Two non-mutually exclusive theories have emerged regarding synaptic regulation underlying memory consolidation during sleep. Studies from Tononi and Cirelli (2006, 2014) have established the synaptic homeostasis hypothesis (Tononi and Cirelli, 2006, 2014) which proposes that neurons form and strengthen synapses during wakefulness as organisms interact with the environment and encode new memories. During sleep, when the encoding process is offline, synapses are downscaled to prevent over-excitation of neurons and improve signal-to-noise ratio and memory performance (Tononi and Cirelli, 2006, 2014). Several studies reporting decreases in dendritic spines, synaptic density, and synaptic markers during sleep in sensory and motor cortical regions provide support for broad synaptic downscaling during sleep (Cirelli, 2005; Maret et al., 2011; de Vivo et al., 2017).

An alternate theory originally proposed by Mueller and Pilzecker and expanded by several groups (McGaugh, 1999) including more recently by Born and colleagues (Rasch and Born, 2013; Dudai et al., 2015), proposes that selective synapses formed during the day are selectively tagged and strengthened during sleep as memory storage is reorganized. Several recent studies support this theory. For example, evidence that sleep deprivation impairs memory strength (Vecsey et al., 2009), and results in decreased dendritic spines (Havekes et al., 2016), supports the hypothesis that selective synapses are strengthened during sleep. In addition, *in vivo* dendritic spine imaging demonstrated that sleep promotes increases in dendritic spines in selective branches of motor cortex neurons following motor learning (Yang et al., 2014), providing further support that sleep strengthens selective synapses formed during recent learning, even in areas where net synaptic downscaling during sleep was reported (Maret et al., 2011; de Vivo et al., 2017).

Despite the growing evidence for synaptic changes during sleep supporting both downscaling and upscaling theories, very few studies have examined dendritic spine regulation during sleep in subcortical areas involved in emotional memory processing. In our recent study, we observed region- and branch-specific synaptic downscaling in the hippocampus of adult mice, supporting the theory of broad but selective synaptic downscaling in this subcortical region (Gisabella et al., 2020). Furthermore, a recent electron microscopy study in adolescent mice provides additional evidence for broad downscaling in this region (Spano et al., 2019). The amygdala is a key subcortical region involved in emotional memory processing and sleep. The amygdala is highly interconnected with the hipppocampus and these two regions work together during emotional memory processing, with the amygdala representing a key region critically invovled in stress regulation and fear memory (Wilensky et al., 2000; Pare et al., 2004; LeDoux, 2007; Rodrigues et al., 2009; Johansen et al., 2011), while the hippocampus is critical for remembering the spatial context of the fearful stimulus. Recent evidence indicates that the amygdala is part of a network together with the hippocampus and prefrontal cortex involved in fear memory consolidation during sleep (Miyawaki and Mizuseki, 2022). Furthermore, the central nucleus of the amygdala (CEA) projects to brainstem REM regulatory regions (Inagaki et al., 1983; Wellman et al., 2022) and recent evidence demonstrates that the amygdala actively participates in the regulation of sleep architecture (Hasegawa et al., 2022). Specifically, dopamine signaling in the basolateral amygdala (BL) is involved in promoting the transition from NREM to REM sleep (Hasegawa et al., 2022). In comparison, the lateral amygdala (LA) serves as a sensory gateway to the amygdala, integrating sensory inputs from thalamic and cortical areas (LeDoux et al., 1990a; Li et al., 1995). Recent studies suggest that synaptic downscaling occurs specifically during REM sleep (Li et al., 2017; Zhou et al., 2020), thus the amygdala may also regulate synaptic processing during sleep through its role in promoting REM sleep.

The amygdala is also critically involved in the regulation emotional behaviors implicated in psychiatric disorders (Bishop et al., 2004). For example, schizophrenia (SZ) and post-traumatic stress disorder (PTSD) display abnormalities in emotional processing as well sleep disturbances and memory dysfunction (Frodl et al., 2002; Sah et al., 2003; Benarroch, 2015; Krystal et al., 2016; Ferrarelli and Tononi, 2017). The amygdala is hyperactive in PTSD (Bremner et al., 2005) and regulates fear and stress alterations in sleep (Wellman et al., 2008, 2013, 2016; Liu et al., 2009). Furthermore, evidence suggests the amygdala is associated with heightened emotional reactivity in insomnia disorders (Baglioni et al., 2010, 2014). Studies in humans describe amygdala hyperreactivity due to sleep loss (Motomura et al., 2017). Interestingly, altered numbers of dendritic spines, which serve as morphological representations of encoded memory, are also consistently reported across several psychiatric disorders (Dorph-Petersen et al., 2009; Sweet et al., 2009; Penzes et al., 2011; Duman and Aghajanian, 2012; Glausier and Lewis, 2013; Licznerski and Duman, 2013; Shelton et al., 2015; MacDonald et al., 2017).

Currently there is no evidence available regarding the effects of sleep or sleep deprivation on dendritic spines in the amygdala. Chronic stress studies have shown that dendritic spines are differentially affected in the amygdala compared to the hippocampus, with dendritic spine density increasing in the amygdala but decreasing in the hippocampus following chronic stress (Vyas et al., 2002). This suggests that memory consolidation may differentially affect dendritic spine morphology between these two regions. We tested the hypothesis that sleep deprivation prevents broad synaptic downscaling in the amygdala. We used viral vector labeling of dendritic spines combined with confocal imaging and three-dimensional (3D) analysis in sleep-deprived and control mice to determine how sleep deprivation affects dendritic spines on neurons in the subregions of the amygdala. Immunohistochemistry and quantitative microscopy for phosphorylated cofilin (pCoflin), a marker involved in the dendritic spine downscaling (Cichon et al., 2012; Calabrese et al., 2014; Havekes et al., 2016; Noguchi et al., 2016) and for growth hormone (GH), a molecule shown to increase dendritic spines and fear memory in the amygdala (Gisabella et al., 2016), were used as a first step in examining the molecular factors involved in synaptic changes during sleep. Specifically, cofilin is involved in cleaving actin filaments during dendritic spine downscaling, and phosphorylation of cofilin decreases its activity (Havekes et al., 2016). Thus, pCofilin levels are predicted to be inverse to spine density measures in sleep deprived mice, whereas GH measures are predicted to positively correlate with spine density measures.

## Methods

### Animals

All mice used in experiments were adult male C57BL/6J mice (available from Jackson Laboratories, Bar Harbor, ME). Animals were maintained on a 12:12 h light-dark cycle with *ad libitum* access to water and food. All animal procedures met National Institutes of Health standards, as outlined in the Guide for the Care and Use of Laboratory Animals, and all protocols were approved by the University of Mississippi Medical Center Institutional Animal Care and Use Committee (IACUC).

### Viral vector injection and sleep deprivation

Adult (3–4 months old) male C57BL/6J mice (6 controls, 6 sleep-deprived mice) received bilateral stereotaxic microinjections of 2 μl of AAV1-CMV-eGFP virus (Vector Biolabs, cat# 7002) in each hemisphere. This number of animals per group was sufficient to detect changes in dendritic spines from sleep deprivation in mice in previous studies from our lab and others (Havekes et al., 2016; Spano et al., 2019; Gisabella et al., 2020). Injections of the AAV-GFP virus under the CMV promoter allowed for the labeling of dendritic branches for spine analysis. The injections targeted the amygdala (BLA) region (stereotaxic coordinates AP:−1.1, ML:3.2, DV:4.1). The virus was infused using a Nanofil 35-gauge stainless steel bevel needle (catalog # NF35BV, World Precision Instruments, Inc., Sarasota, FL) attached to a 10μl Nanofil syringe (Hamilton Company, Reno, NV). Hamilton syringes were mounted in a stereotaxic barrel holder, and the rate of virus delivery was controlled by an automated syringe pump (Harvard Apparatus, Holliston, MA). After 6 weeks, animals were divided randomly into two cohorts of six mice each (6 controls, 6 sleep-deprived mice). The Pinnacle automated sleep deprivation system (Cat# 9000-K5-S) which simulates gentle handling was used for 5 h of sleep deprivation from lights on (6 AM) to 11 AM. This system consists of a cylindrical housing chamber with a bar that continuously rotates at 5 rpm and randomly reversed direction every 10–30 s, which prevents animals from sleeping. All animals were housed in the cylindrical chambers beforehand to adapt to the environment and control animals were housed in the same chambers but without the rotating bar. A researcher visually verified that the bar rotated at all times and that mice did not use alternative strategies to avoid the bar and sleep. This system has been established by prior studies to effectively reduce sleep as measured with EEG recordings in rats and mice (Hines et al., 2013; Burgdorf et al., 2019; Aguilar et al., 2020; Yuan et al., 2021). Both control and sleep-deprived mice were sacrificed at 11 AM (5 h after lights on in the 12:12 light cycle) and processed for quantification of dendritic spines. This time point 5 h into the light cycle is identical to previous studies from our lab and others (Havekes et al., 2016; Gisabella et al., 2020). Brains were then analyzed for viral vector expression and dendritic spines as in our published and preliminary studies (Gisabella et al., 2020).

### Tissue processing

Mice were deeply anesthetized with isoflurane and perfused with 0.1M phosphate-buffered saline (PBS) containing 10% formalin. Brains were removed and cryoprotected in 30% sucrose in 0.1M PBS (pH 7.4), then sectioned into coronal 40 μm serial sections using a freezing microtome (American Optical 860, Buffalo, NY) and stored in 0.1 Molar phosphate buffer with 0.1% NaAzide. Sections were mounted on gelatin-coated slides and coverslipped using Dako mounting media (S3023, Dako, North America, Carpinteria, CA) to quantify dendritic spine density from images captured using confocal microscopy.

#### Immunocytochemistry (mouse samples)

Free-floating mouse brain tissue sections were carried through antigen retrieval in citric acid buffer (0.1 M citric acid, 0.2 M Na_2_HPO_4_) heated to 80°C for 30 min, and incubated in the rabbit primary antibody anti-pCofilin (Ser3) (cat#3311, Cell Signaling) (1:1000 μl) or rabbit anti-GH1 (Protein Tech Lab, cat# 55243–1-AP) (1:500 μl) for 48 h at 4°C, and subsequently in biotinylated secondary antibody (goat anti-rabbit IgG; 1:500; Vector Labs, Inc. Burlingame, CA), followed by streptavidin conjugated with horse-radish peroxidase for 2 h (1:5000 μl, Zymed, San Francisco, CA), and, finally, in nickel-enhanced diaminobenzidine/ peroxidase reaction (0.02% diaminobenzidine, Sigma-Aldrich, 0.08% nickel-sulfate, 0.006% hydrogen peroxide in PB). All solutions were made in PBS with 0.2% Triton X (PBS-Tx) unless otherwise specified. Immunostained sections were mounted on gelatin-coated glass slides, dehydrated in a gradient ethanol series, coverslipped, and coded for blinded quantitative analysis. All sections included in the study were processed simultaneously within the same session to avoid procedural differences. Omission of the primary or secondary antibodies did not result in detectable signal.

### Confocal imaging

A Zeiss LSM 880 confocal microscope system interfaced with Zen imaging software (ZEN 2.3 SP1) was used to acquire 3D image stacks of dendritic branches from amygdala neurons in sections from control and sleep-deprived mice. All slides used for confocal imaging were coded for blind analysis. Images of 40 μm-thick sections were acquired, with a z-step of 0.5 μm using a 63x oil immersion objective (numerical aperture 1.4 DIC M27; pixel size, 0.10 × 0.10 μm) similar to the method described in our previous study (Gisabella et al., 2016). For dendritic spine quantification, confocal microscopy images were analyzed using Neurolucida 360 software with Autospine to measure spine density in the amygdala cells using an approach previously described (Gisabella et al., 2016). Dendritic spines were sampled in the Lateral (LA), Basolateral (BL), and Central (CEA) areas of the amygdala by an investigator who was blinded to the treatment group. The complex organization of neurons and their dendritic branches in the amygdala, together with the variation in injection sites within each animal made it challenging to follow branches from individual neurons and to establish sampling strategies. Therefore, we collected confocal images of all clearly visible viral vector labeled dendritic branches within the lateral, basolateral, or central nuclei using 25 × 25 μm confocal imaging windows. The minimal viral spread allowed for the feasibility of this approach. Spine density, shape, and volume were analyzed using Neurolucida 360 with semiautomated analysis from 3D confocal image stacks. We quantified the numbers of mushroom, thin, and stubby spines in each branch segment, and the spine head diameter and volume of each spine.

### Dendritic spine quantification

For dendritic spine quantification, confocal microscopy images were analyzed using Neurolucida 360 software with Autospine to measure spine density as described in our previous study (Gisabella et al., 2016). Spines were grouped into thin spines, stubby spines, and mushroom spines automatically by the Neurolucida 360 software based on the spine head to neck diameter ratio (1.1), length-to-head ratio (2.5) mushroom head size (0.35 μm), and filopodia length (3 μm) according to previously established criteria (Rodriguez et al., 2008). Spine density, shape, and volume were quantified using Neurolucida 360 with semiautomated analysis from 3D confocal image stacks in an unbiased manner.

#### Microscopy data collection

In mouse brain samples, serial sections containing the amygdala were quantified using a Leica microscope interfaced with Bioquant Nova Prime v6.0, (R&M Biometrics, Nashville, Tennessee) for pCofilin and using a Zeiss Axioskop 40 interfaced with StereoInvestigator v2021.1.3 for growth hormone. Borders of each region were defined according to the Allen Brain Atlas and traced under 4x magnification. Each traced region was systematically scanned through the full x, y, and z-axes under 40x magnification to count each pCofilin immunoreactive (IR) cell and under 20x magnification to count each GH-IR cell.

### Statistical analysis

Densities of dendritic spines were calculated as spines per dendrite segment length in micrometers. Numerical densities of pCofilin cells were calculated as N_d_ = ∑N / ∑V where N = sum of all pCofilin or GH immunoreactive cells counted in each region for each animal, and V is the volume of each region per animal, calculated as V= ∑a • z, where z is the thickness of each section (30 μm) and a is area in μm^2^. For all statistical tests, the significance threshold was *p* ≤ 0.05. Non-paramatric (Wilcoxon-Mann-Whitney) tests were used to compare population estimates for control and sleep-deprived groups as data were not normally distributed, and were followed by Bonferroni *post hoc* correction. Box plots were used to depict the data for each group from *n* = 6 control and *n* = 6 sleep-deprived animals.

## Results

### Lower density of basolateral amygdala dendritic spines in sleep deprived mice

**FIGURE 1:**
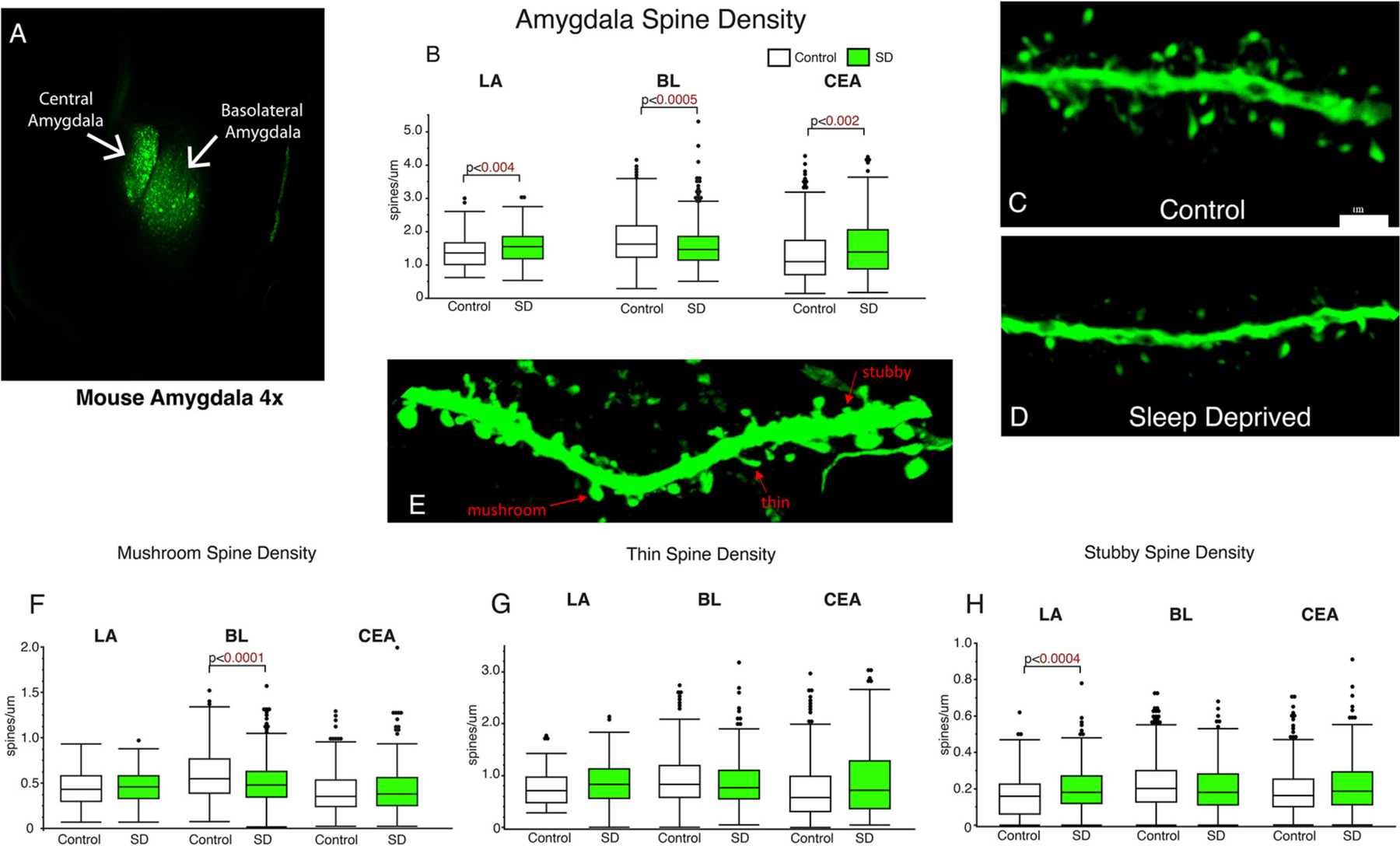
Dendritic spines in the basolateral amygdala (BL) are lower in sleep-deprived mice. AAV viral vector was used to label dendritic processes **(A)**. Decreased density of dendritic spines was observed in the BL amygdala in sleep-deprived mice (n_mice_ = 6; n_dendrites_ = 383) compared to control mice (n_mice_ = 6; n_dendrites_ = 450) **(B)**. In contrast, density of dendritic spines in the LA of sleep-deprived mice (n_mice_ = 5; n_dendrites_ = 167) was increased in comparison to control mice (n_mice_ = 5; n_dendrites_ = 105) **(B)**. A similar increase in density was observed in the CEA of sleep-deprived mice (n_mice_ = 5; n_dendrites_ = 222) compared to control mice (n_mice_ = 5; n_dendrites_ = 239). Representative confocal micrograph of a 10 μm branch segment from the BL of a sleep-deprived mouse **(D)**, with fewer spines than a representative branch segment from a control mouse **(C)**. **(E)** Representative confocal image depicting examples of thin, mushroom, and stubby spines. Dendritic spine density was decreased in mushroom spines in the BL of sleep-deprived mice (n_mice_ = 6; n_dendrites_ = 383) compared to control mice (n_mice_ = 6; n_dendrites_ = 450) **(F).** Mushroom spines from the LA of sleep-deprived mice (n_mice_ = 5; n_dendrites_ = 167) were not significantly different from control mice (n_mice_ = 5; n_dendrites_ = 105) **(F)**. Similarly, no differences were observed in CEA mushroom spines from sleep deprived mice (n_mice_ = 5; n_dendrites_ = 222) compared to control mice (n_mice_ = 5; n_dendrites_ = 239). No difference in density of thin spines was observed in the LA of sleep-deprived mice (n_mice_ = 5; n_dendrites_ = 167) compared to control mice (n_mice_ = 5; n_dendrites_ = 105) **(G)**, or in thin spines from the CEA of sleep-deprived mice (n_mice_ = 5; n_dendrites_ = 222) compared to control mice (n_mice_ = 5; n_dendrites_ = 239) **(G)**. Similarly, no difference was observed in the density of thin spines in the BL of sleep deprived mice (n_mice_ = 6; n_dendrites_ = 383) compared to control mice (n_mice_ = 6; n_dendrites_ = 450). Increased density of stubby spines was observed in the LA of sleep-deprived mice (n_mice_ = 5; n_dendrites_ = 167) compared to control mice (n_mice_ = 5; n_dendrites_ = 105) **(H)**. No difference was observed in the CEA of sleep-deprived mice (n_mice_ = 5; n_dendrites_ = 222) compared to control mice (n_mice_ = 5; n_dendrites_ = 239), or in the density of stubby spines in the BL of sleep deprived mice (n_mice_ = 6; n_dendrites_ = 383) compared to control mice (n_mice_ = 6; n_dendrites_ = 450) **(G)**. Box plots depict values for each group, statistical significance was determined using the Wilcoxon-Mann-Whitney test with Bonferroni correction for multiple comparisons.

Dendritic spines were quantified from amygdala subregions including the LA, BL, and CEA (Figure 1). We observed significantly decreased dendritic spine density in the BL of sleep deprived mice (*p* < 0.0005; Figure 1B) compared to the littermates that were permitted to sleep undisturbed (control mean: 1.76, SD mean: 1.59, 10.15 % decrease). In comparison, dendritic spine density was increased in the LA (control mean: 1.39, SD mean: 1.55, 10.88 % increase) and CEA (control mean: 1.35, SD mean: 1.55, 13.8 % increase) of sleep deprived mice (Figure 1B). Decreased density of dendritic spines in the BL was driven by mushroom spines (*p* < 0.0001; Figure 1F) (control mean: 0.57, SD mean: 0.50, −13.01 % decrease). In contrast, the increase in dendritic spine density in sleep-deprived mice in the LA was observed in stubby spines (LA: *p* < 0.0004; Figure 1H) (Control mean: 0.17, SD mean: 0.21, 21.05 % increase).

### Subregion and spine type specific increases in neck length of dendritic spines in sleep deprived mice

**FIGURE 2:**
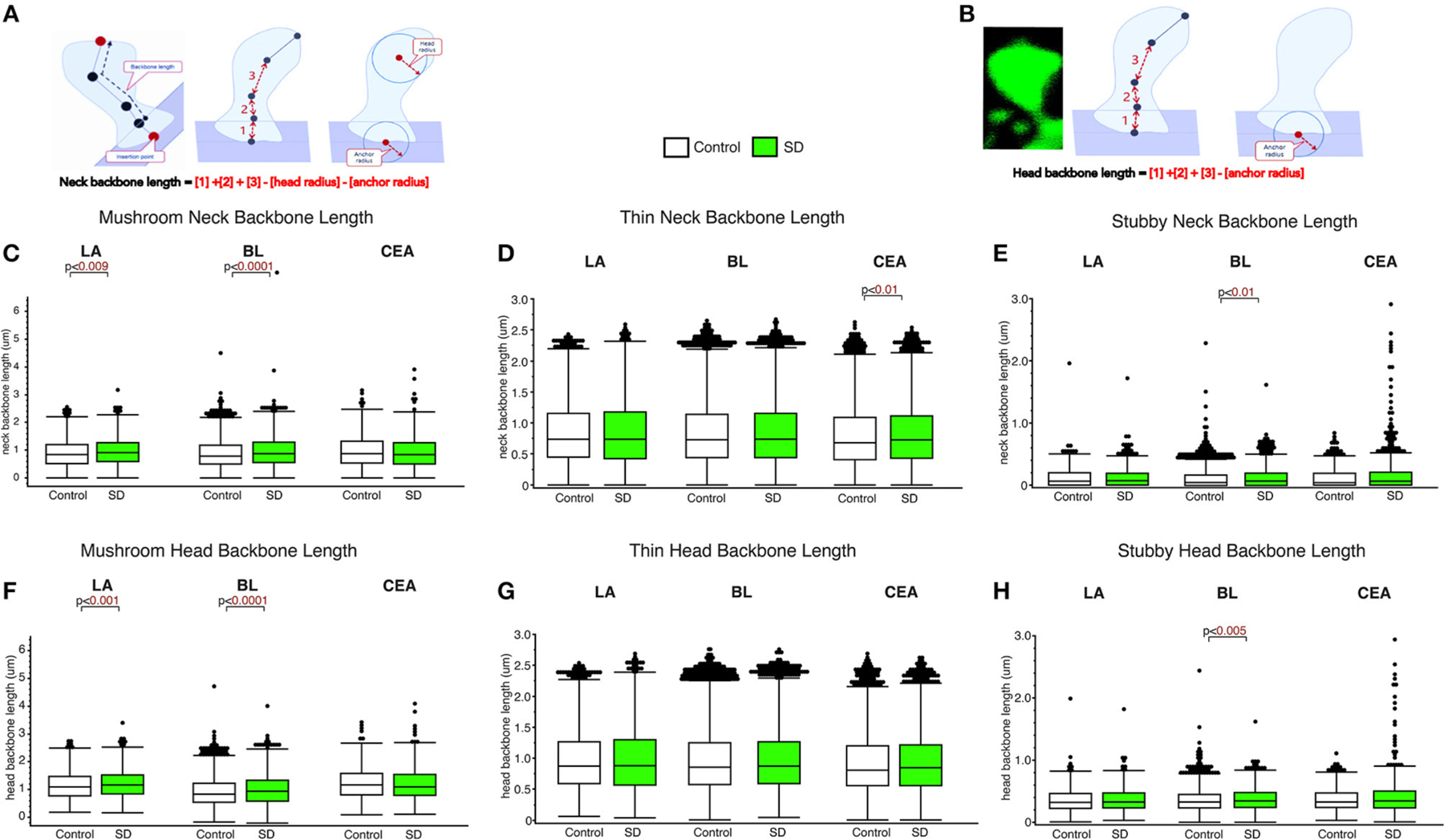
Differential changes in neck backbone and head backbone length dendritic spines in sleep deprived mice. Neurolucida 360 was used to obtain measures of dendritic spine neck backbone length and head backbone length from confocal images. The diagram **(A, B)** depicts neck backbone length measured as the distance from the insertion point to the center of the spine head minus the head radius and anchor radius **(A)**. Head backbone length is measured as the distance from the insertion point to the center of the spine head minus the anchor radius **(B)**. Neck backbone length was significantly greater in mushroom spines from the LA of sleep-deprived mice (n_mice_ = 5; n_spines_ = 1,339) compared to control mice (n_mice_ = 5; n_spines_ = 1,217) **(C)**. A similar increase was observed in the BL of sleep-deprived mice (n_mice_ = 6; n_spines_ = 3,143) compared to control mice (n_mice_ = 6; n_spines_ = 5,385) for mushroom spine backbone length **(C)**. No significant difference was observed for neck backbone length of mushroom spines in the CEA of sleep deprived mice (n_mice_ = 5; n_spines_ = 1,543) compared to control mice (n_mice_ = 5; n_spines_ = 1,768). In comparison, neck backbone length in thin spines was not altered in the LA of sleep-deprived mice (n_mice_ = 5; n_spines_ = 2,525) compared to control mice (n_mice_ = 6; n_spines_ = 2,046) or in the BL of sleep-deprived mice (n_mice_ = 5; n_spines_ = 5,645) compared to control mice (n_mice_ = 5; n_spines_ = 9,079). Neck backbone length was increased in thin spines from the CEA in sleep-deprived mice (n_mice_ = 5; n_spines_ = 3,207) compared to control mice (n_mice_ = 5; n_spines_ = 3,449) **(D)**. Neck backbone length was greater in stubby spines from the BL of sleep deprived mice (n_mice_ = 6; n_spines_ = 1,343) compared to control mice (n_mice_ = 6; n_spines_ = 2,230). No difference in neck backbone length was observed in the CEA of sleep-deprived mice (n_mice_ = 5; n_spines_ = 878) compared to control mice (n_mice_ = 5; n_spines_ = 871) **(E)**, or in stubby spines in the LA of sleep-deprived mice (n_mice_ = 5; n_spines_ = 618) compared to control mice (n_mice_ = 5; n_spines_ = 469). Similar changes were observed for head backbone length measures, with increased length in mushroom spines from sleep-deprived mice vs. controls in the LA and BL areas and for stubby spines in the CEA **(F–H)**. Box plots depict values for each group, statistical significance was determined using the Wilcoxon-Mann-Whitney test with Bonferroni correction for multiple comparisons.

The LA and BL displayed significantly greater neck backbone length of mushroom spines in sleep-deprived mice vs. control mice (Figure 2; LA: *p* < 0.009 and BL: *p* < 0.0001) (LA: control mean: 0.88, SD mean: 0.95, 7.70% increase; BL: control mean: 0.88, SD mean: 0.95, 7.70% increase). In comparison, thin spines displayed increased neck backbone length in sleep deprived mice in the CEA (Figure 2; *p* < 0.01) (control mean: 0.78, SD mean: 0.82, 5.00 % increase) and stubby spines displayed greater neck backbone length in sleep deprived mice in the BL (*p* < 0.01), Figure 2E (control mean: 0.35, SD mean: 0.37, 5.56 % increase). Similar changes were observed regarding head backbone length (Figures 2F–H). Mushroom head backbone length was greater in sleep-deprived mice vs. controls in the LA and BL areas (LA: *p* < 0.001 and BL: *p* < 0.0001, Figure 2F) (LA: control mean: 1.15, SD mean: 1.21, 5.10% increase; BL: control mean: 1.14, SD mean: 1.20, 5.13% increase). Stubby dendritic spines in the BL amygdala displayed significantly greater head backbone length (*p* < 0.005, Figure 2H) in sleep-deprived mice vs. controls (control mean: 0.35, SD mean: 0.37, 5.56% increase).

### Subregion and spine type specific changes in spine head and neck diameter in sleep deprived mice

**FIGURE 3:**
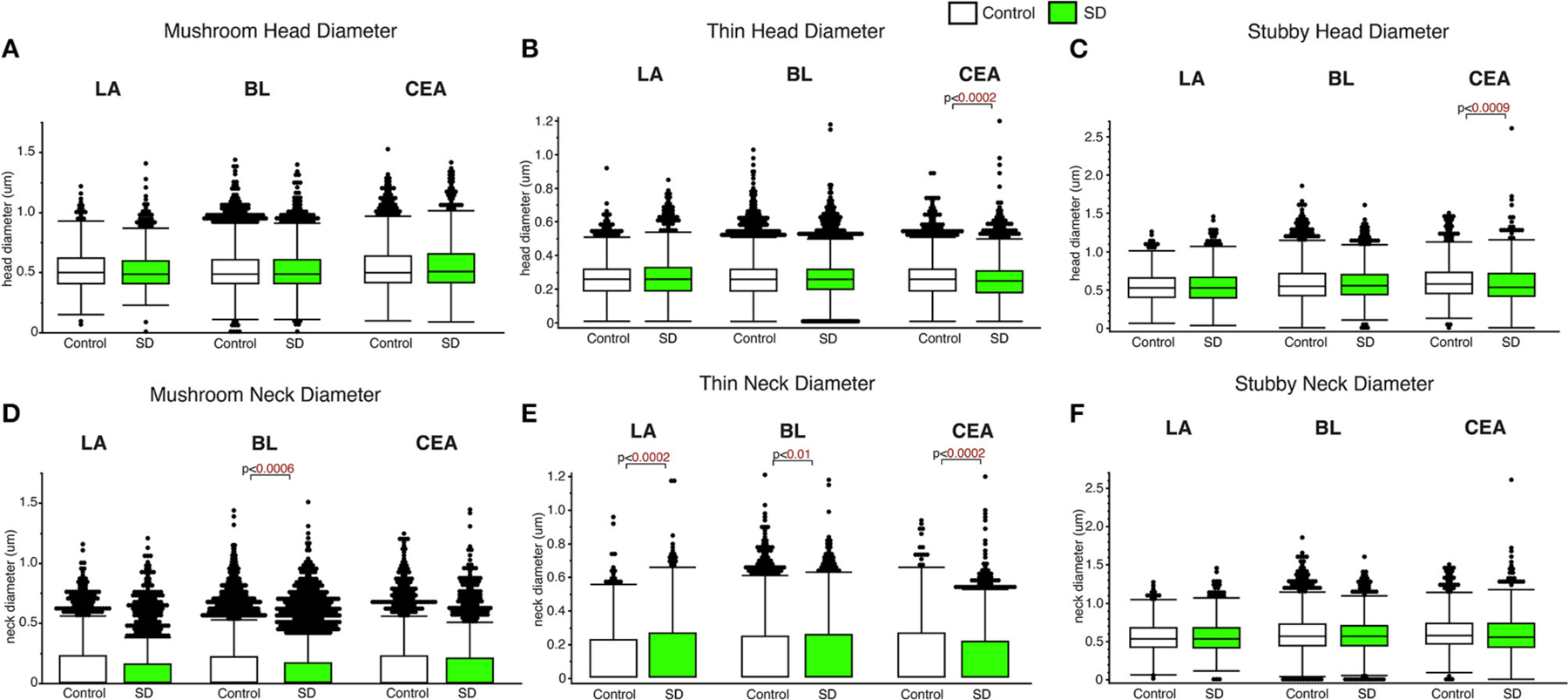
Differential changes of head and neck diameter across dendritic spine types during sleep. Mushroom spine head diameter was not significantly different between sleep-deprived and control mice in the LA (Control: n_mice_ = 5; n_spines_ = 1,217; SD: n_mice_ = 5; n_spines_ = 1,339), BL (Control: n_mice_ = 6; n_spines_ = 5,385; SD: n_mice_ = 6; n_spines_ = 3,143) or CEA (Control: n_mice_ = 5; n_spines_ = 1,768; SD: n_mice_ = 5; n_spines_ = 1,543) **(A)**. Thin spine head diameter was selectively decreased in the CEA of sleep-deprived mice (Control: n_mice_ = 5; n_spines_ = 3,449; SD: n_mice_ = 5; n_spines_ = 3,207) **(B)**, with no significant differences in the LA (Control: n_mice_ = 5; n_spines_ = 2,046; SD: n_mice_ = 5; n_spines_ = 2,525) or BL (Control: n_mice_ = 6; n_spines_ = 9,079; SD: n_mice_ = 6; n_spines_ = 5,645) **(B)**. A similar selective decrease was observed for stubby spines in the CEA (Control: n_mice_ = 5; n_spines_ = 871; SD: n_mice_ = 5; n_spines_ = 878), with no significant changes in the LA (Control: n_mice_ = 5; n_spines_ = 469; SD: n_mice_ = 5; n_spines_ = 618) or BL (Control: n_mice_ = 6; n_spines_ = 2,230; SD: n_mice_ = 6; n_spines_ = 1,343) **(C)**. Spine neck diameter was significantly lower in mushroom spines in the BL of sleep deprived mice, with no changes in mushroom spines in the LA or CEA **(D)**. In contrast, neck diameter for thin spines was significantly greater in the LA and BL of sleep-deprived mice and lower in the CEA **(E)**. Neck diameter for stubby spines was not altered **(F)**. Box plots depict values for each group, statistical significance was determined using the Wilcoxon-Mann-Whitney test with Bonferroni correction for multiple comparisons.

Decreased spine head diameter was observed in the CEA for thin (*p* < 0.0002; Figure 3) and stubby (*p* < 0.009; Figure 3C) spines in sleep deprived mice compared to control mice (thin: control mean: 0.26, SD mean: 0.25, 3.92% decrease; stubby: control mean: 0.61, SD mean: 0.58, 5.04 % decrease). No changes in spine head diameter were detected in thin or stubby spines the LA or BL and no changes were observed in mushroom spine head diameters in any area (Figures 3A–C). In comparison, significantly decreased spine neck diameter was observed in the mushroom spines in the BL amygdala (*p* < 0.0006; Figure 3D) (control mean: 0.14, SD mean: 0.13, 7.41 % decrease), as well as in thin spines in the CEA (thin spines: *p* < 0.0002; Figure 3E) (control mean: 0.13, SD mean: 0.12, 8.92% decrease). In contrast, we observed increased neck diameter in thin dendritic spines located in the LA (*p* < 0.0002; Figure 3E) and BL (*p* < 0.01; Figure 3E) amygdala in sleep deprived mice (LA: Control mean: 0.12, SD mean: 0.14, 15.38% increase; BL: Control mean: 0.12, SD mean: 0.13, 8.00% increase).

### Spine subtype and amygdala region specific changes in surface area and volume in sleep deprived mice

**FIGURE 4:**
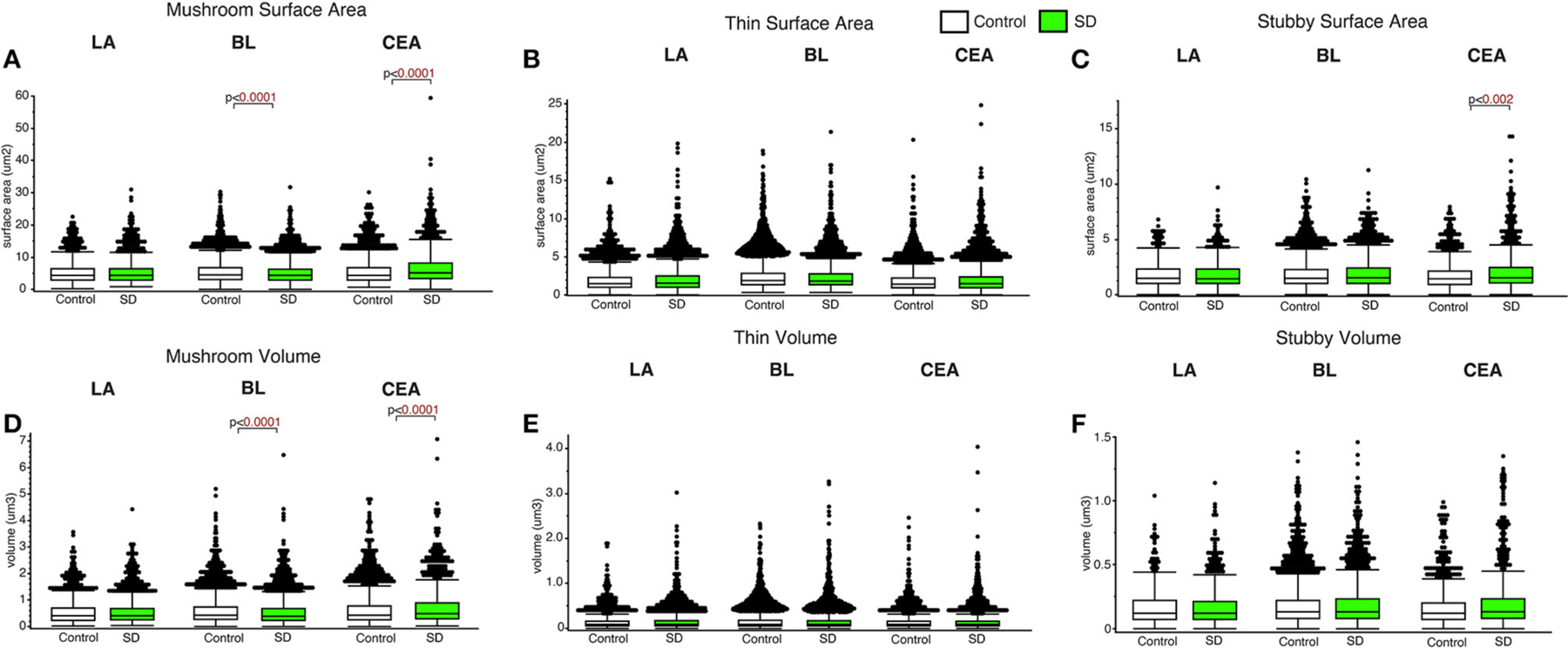
Dendritic spine surface area and volume differences between sleep-deprived and control mice. Dendritic spine surface area was significantly lower in mushroom spines from sleep-deprived mice in the BL compared to control mice (Control: n_mice_ = 6; n_spines_ = 5,385; SD: n_mice_ = 6; n_spines_ = 3,143) **(A)**. Spine surface area was significantly greater in mushroom spines from sleep-deprived mice in the CEA (Control: n_mice_ = 5; n_spines_ = 1,768; SD: n_mice_ = 5; n_spines_ = 1,543), and no differences were observed for mushroom spine surface area in the LA (Control: n_mice_ = 5; n_spines_ = 1,217; SD: n_mice_ = 5; n_spines_ = 1,339) **(A)**. Thin spine surface area was not significantly altered in the LA (Control: n_mice_ = 5; n_spines_ = 2,046; SD: n_mice_ = 5; n_spines_ = 2,525), CEA (Control: n_mice_ = 5; n_spines_ = 3,449; SD: n_mice_ = 5; n_spines_ = 3,207) or BL (Control: n_mice_ = 6; n_spines_ = 9,079; SD: n_mice_ = 6; n_spines_ = 5,645) **(B)**. The surface area of stubby spines was significantly greater in spines from the CEA (Control: n_mice_ = 5; n_spines_ = 871; SD: n_mice_ = 5; n_spines_ = 878) of sleep-deprived mice, with no differences observed from stubby spines in the LA (Control: n_mice_ = 5; n_spines_ = 469; SD: n_mice_ = 5; n_spines_ = 618) or BL (Control: n_mice_ = 6; n_spines_ = 2,230; SD: n_mice_ = 6; n_spines_ = 1,343) **(C)**. Spine volumes displayed largely similar changes, with significantly lower volume in the BL and greater volume in the CEA for mushroom spines in sleep-deprived mice **(D)**, no significant difference in thin **(E)**, and greater volume of stubby spines in the CEA of sleep-deprived mice **(F)**. Box plots depict values for each group, statistical significance was determined using the Wilcoxon-Mann-Whitney test with Bonferroni correction for multiple comparisons.

Decreased dendritic spine surface area was observed in the BL in sleep deprived mice compared to control mice for mushroom spines (*p* < 0.0001; Figure 4) (control mean: 5.39, SD mean: 5.05, −6.51% decrease). In comparison, increased surface area was observed in sleep deprived mice for mushroom spines located in CEA (*p* < 0.0001; Figure 4A) (control mean: 5.45, SD mean: 6.46, 16.97 % increase). Decreased dendritic spine head volume was observed specifically in mushroom spines in sleep deprived mice in the BL amygdala (*p* < 0.0001; Figure 4D) (control mean: 0.57, SD mean: 0.53, −7.27 % decrease). In contrast, spine volume was significantly increased in sleep deprived mice in mushroom spines in the CEA (*p* < 0.0001; Figure 4D) (control mean: 0.62, SD mean: 0.69, 10.69 % increase) and stubby spines in the CEA (*p* < 0.002; Figure 4F) (control mean: 1.79, SD mean: 2.10, 15.94% increase).

### Lower densities of pCofilin and growth hormone immunoreactive cells in sleep deprived mice

**FIGURE 5:**
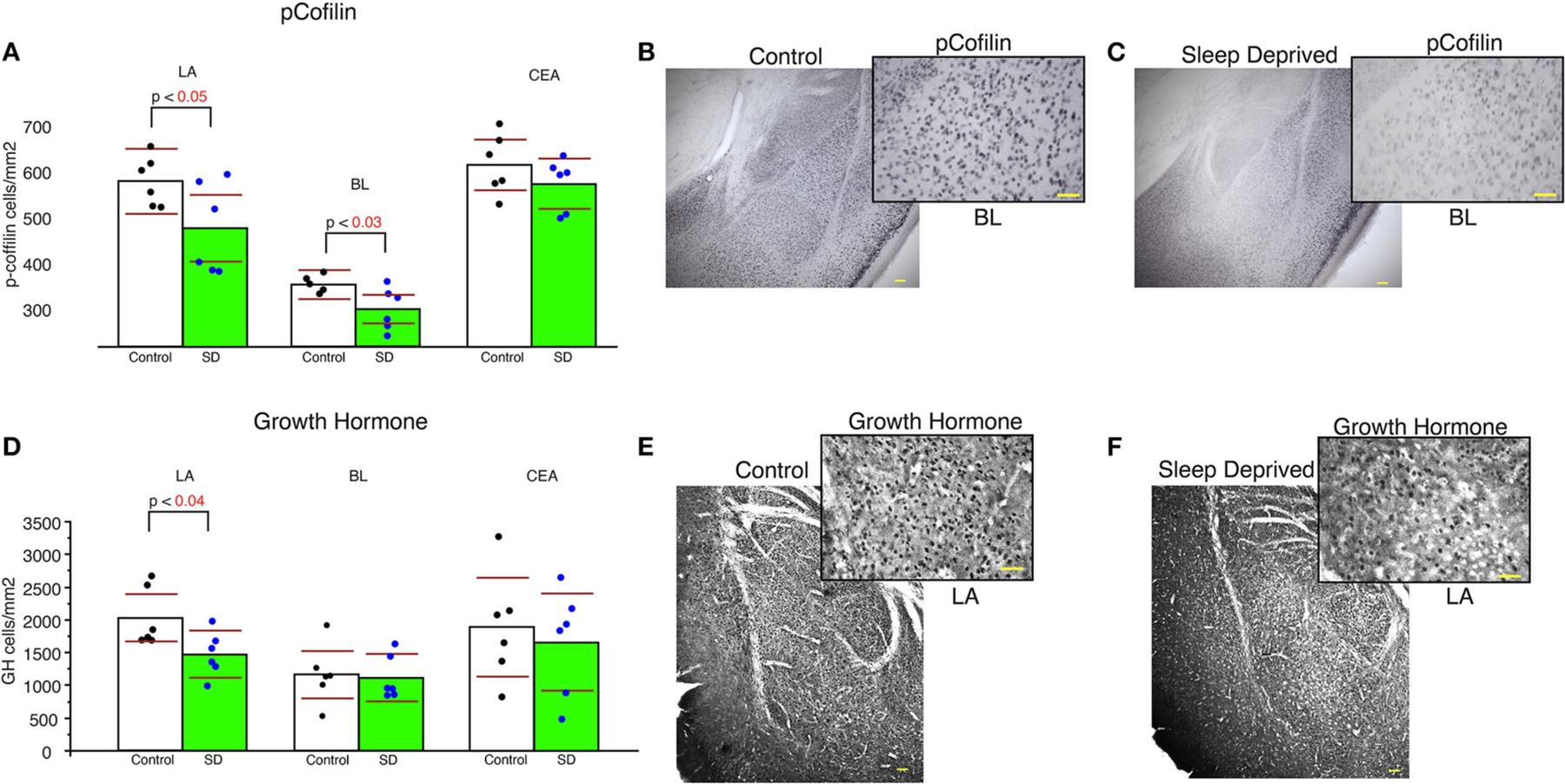
Lower density of pCofilin and GH immunoreactive cells in the amygdala of sleep deprived mice. Sleep-deprived mice displayed significantly reduced numerical density of pCofilin immunoreactive cells in the lateral and basolateral amygdala nuclei, but not in the central nucleus compared to control mice **(A)**. Representative 5x and 40x images of pCofilin labeling in the amygdala from control **(B)** and SD mice **(C)**. 40x magnification inserts depict pCofilin labeling in the BL. Decreased density of GH immunoreactive cells was also detected in the LA of sleep deprived mice **(D)**, but not in the BL or CEA. Representative 5x and 40x images of GH labeling in the amygdala from control **(E)** and SD mice **(F)**. 40x magnification inserts depict GH labeling in the LA. Scale bars in yellow = 100 μm for 5x images, 50 μm for 40x inserts. All graphs reflect the mean for each group with *n* = 6 control and *n* = 6 sleep-deprived animals. Bar graphs depicting mean and 95% confidence interval of the density of immunoreactive cells. Each dot represents the value for an individual mouse calculated as density of immunoreactive cells per area (cells/mm^2^).

Sleep-deprived mice displayed significantly lower density of pCofilin immunoreactive cells in the LA and BL amygdala nuclei compared to control mice (LA: *p* < 0.05 and BL: *p* < 0.03, Figure 5A). In comparison, no significant changes in density of pCofilin IR cells was detected in the CEA (Figure 5). A similar decrease was observed for densities of GH IR cells in the LA of sleep deprived mice (Figure 5D). No changes were detected for GH densities in the BL or CEA (Figure 5D).

## Discussion

Our results point to region and spine type specific dendritic spine changes during sleep in the amygdala. In summary, we observed evidence for selective strengthening of mushroom spines in the BL amygdala during sleep together with downscaling of thin and stubby spines in the LA and CEA regions. Despite several studies describing the effects of sleep deprivation in cortical areas and in the hippocampus (Yang et al., 2014; Havekes et al., 2016; Spano et al., 2019; Gisabella et al., 2020), our findings represent the first evidence for the effect of sleep deprivation on dendritic spines in the amygdala. Several lines of evidence point to a key role of the amygdala in sleep and emotional memory processing. For example, the amygdala is selectively activated during rapid eye movement sleep (REM) and is involved in consolidation of emotional memories (Goldstein and Walker, 2014; Genzel et al., 2015; Murkar and De Koninck, 2018). Furthermore, studies in human subjects indicate that REM sleep is essential for next-day social and emotional memory functioning (Goldstein and Walker, 2014). In particular, sleep is involved in regulating emotional reactivity, and amygdala function is dysregulated in sleep deprived subjects (Yoo et al., 2007), with sleep loss consistently contributing to emotional instability (Horne, 1985; Dinges et al., 1997). Taken together, our findings suggest that our evidence for upscaling in the BL amygdala may reflect the role of this area in consolidation of emotional memory during sleep through strengthening of mushroom spines typically associated with more stable, long-term memories, which may represent the convergence of sensory inputs with emotional valence in this area.

### Differential effects of sleep deprivation on basolateral amygdala vs. lateral and central amygdala spines

We observed differential effects of sleep deprivation between amygdala nuclei. The selective decrease of mushroom spine density in the BL of sleep deprived mice, together with the increased neck length and decreased surface area and volume of these spines all point to upscaling in the BL during sleep. This is in sharp contrast to reports of synaptic downscaling during sleep reported in cortical regions and in the hippocampus (Cirelli, 2005; Maret et al., 2011; de Vivo et al., 2017; Spano et al., 2019; Gisabella et al., 2020). The specificity of these changes for mushroom spines supports strengthening of spines associated with long term memory storage during sleep. Furthermore, shorter spine neck length (as measured by head backbone length and neck backbone length) for mushroom spines in the BL of control mice provides additional support for synaptic upscaling, as shorter neck length is associated with faster synaptic transmission and greater synaptic strength (Araya et al., 2014). Similarly, greater spine surface area and volume in mushroom spines in the BL point to synaptic upscaling during sleep, as greater spine volume and surface area is associated with synaptic strength and density of glutamate receptors (Harris and Stevens, 1989; Noguchi et al., 2005; Kopec et al., 2007; Borczyk et al., 2019). Spine neck diameter is reported to be a key indicator of synaptic transmission as narrow neck diameters impede molecular diffusion from the spine head to the dendrite (Noguchi et al., 2005; Adrian et al., 2014, 2017). Furthermore, spine neck diameter has been reported to be positively correlated with spine head volume (Arellano et al., 2007). Our observed decrease of neck diameter in mushroom spines in the BL amygdala of sleep deprived mice suggests that spine neck diameter increases in these spines during sleep, thus contributing to enhanced diffusion and synaptic strength. Similar changes were observed for neck diameters of thin spines in the CEA, pointing to synaptic strengthening in this area.

In contrast, our findings in the LA and CEA point to synaptic downscaling in these areas. Greater densities of thin and stubby spines in sleep deprived mice in these regions indicate that downscaling selectively occurs in these two spine types. Greater neck diameters for thin spines in the LA and BL of sleep deprived mice suggests decreased diffusion for these spines during sleep, and thus weakened synaptic transmission. What may be behind these region-specific effects of sleep deprivation on amygdalar dendritic spines? Differential connectivity may contribute to the divergent effect of sleep deprivation between the BL vs. the LA and CEA areas. The LA receives sensory inputs from thalamic and cortical areas, serving as the sensory gateway to the amygdala (LeDoux et al., 1990a; Li et al., 1995). In comparison, the BL receives multimodal sensory inputs (LeDoux et al., 1990b; Uwano et al., 1995; Maren et al., 1996; Wilensky et al., 1999; LeDoux, 2000; Lucas et al., 2016; Hintiryan and Dong, 2022) as well as dopaminergic input from the VTA (Lutas et al., 2019; Tang et al., 2020), and noradrenergic input from the locus coeruleus (McCall et al., 2017), as well as input from the LA (Pitkanen et al., 1995). In turn, the BLA sends information to several areas including the CEA (Tovote et al., 2016; Hintiryan and Dong, 2022), the medial prefrontal cortex (McDonald, 1992; Cunningham et al., 2002; Cheriyan et al., 2016), the orbitofrontal cortex (Lichtenberg et al., 2017), the nucleus accumbens (Stuber et al., 2011; Wang et al., 2020). It is also the amygdala region that sends the largest amount of projections to areas CA1 and CA3 of the hippocampus (Benes and Berretta, 2000; Pitkanen et al., 2000). Within this context, our data suggest that synapses in the LA potentially formed from sensory information encoded during wakefulness are broadly downscaled during sleep, whereas synapses in the BL, where multimodal sensory information converges with information from areas involved in contextual representations and reward processing to form long-lasting representations (LeDoux et al., 1990b; Maren et al., 1996; Wilensky et al., 1999; LeDoux, 2000), are upscaled during sleep. These upscaled synapses in the BL may in turn contribute hard-wire contextual and multimodal sensory information with fear or reward response processes, resulting in heightened fear, anxiety or addiction related behaviors. Evidence for increased BL activation during REM sleep supports this hypothesis (Corsi-Cabrera et al., 2016; Hasegawa et al., 2022). Furthermore, the BL amygdala is at the hub of interconnected circuits activated during the replay of emotional memories during sleep (Chen and Wilson, 2017; Girardeau et al., 2017; Miyawaki and Mizuseki, 2022), suggesting that re-activation of BL synapses promotes strengthening of emotionally significant information in this region during sleep while broader sensory inputs in the LA are downscaled to make space for new connections, similar to the downscaling reported in cortical areas and the CA1 hippocampus (Cirelli, 2005; Maret et al., 2011; de Vivo et al., 2017; Spano et al., 2019; Gisabella et al., 2020). Sensory information in the LA that is transferred to long-term storage may occur in selective sets of synapses and/or may be transferred to the BL or other areas during sleep. Sleep deprivation may also allow more thin and stubby spines to form in response to additional environmental stimuli during the extended waking period, particularly in the LA. These excess spines may contribute to increased impulsivity and risk-taking behavior reported in subjects with decreased sleep (Demos et al., 2016; Brunet et al., 2020).

### Association of cofilin activity with decreased basolateral amygdala spines in sleep deprived mice

We analyzed neurons expressing pCofilin as a first step in examining molecular pathways that may be involved in dendritic spine changes in sleep deprived mice. Cofilin is modulated by synaptic plasticity (Chen et al., 2007; Pontrello et al., 2012) and is localized in the postsynaptic density where it is believed to function as a key regulator of actin dynamics regulating spine morphology and spine length (Andrianantoandro and Pollard, 2006; Hotulainen et al., 2009). Previous studies suggest that suppression of cofilin activity by phosporylation is important for the stabilization of mature spines, as this prevents cofilin from participating in downregulating these spines by cleaving spine actin filaments (Shi et al., 2009). Furthermore, a recent study demonstrated that 5 h of sleep deprivation results in reduced phosphorylation of cofilin in the hippocampus, which results in greater cofilin activity and in turn enhanced (Havekes et al., 2016). In turn, suppressing cofilin activity prevented the sleep deprivation induced reduction of dendritic spines in the hippocampus (Havekes et al., 2016). Our data showing decreased density of pCofilin neurons in the BL and LA areas of sleep deprived mice indicate increased cofilin activity in sleep deprived mice. Taken together with decreased density of mushroom spines in the BL, this suggests that cofilin activity is enhanced in sleep dperived mice due to reduced phosphorylation of cofilin, and this promotes cofilin cleavage of actin filaments in spines, contributing to synaptic downscaling. Reduced density of pCofilin in the LA area despite decreased spine density in this region may point to subtype specific changes in dendritic spines that are not detected in our broad sampling. Future studies into memory trace specific and neuronal subtype specific dendritic spine changes will provide insight into this process.

### Contrasting effects of sleep deprivation on amygdalar vs. hippocampal spines

**FIGURE 6:**
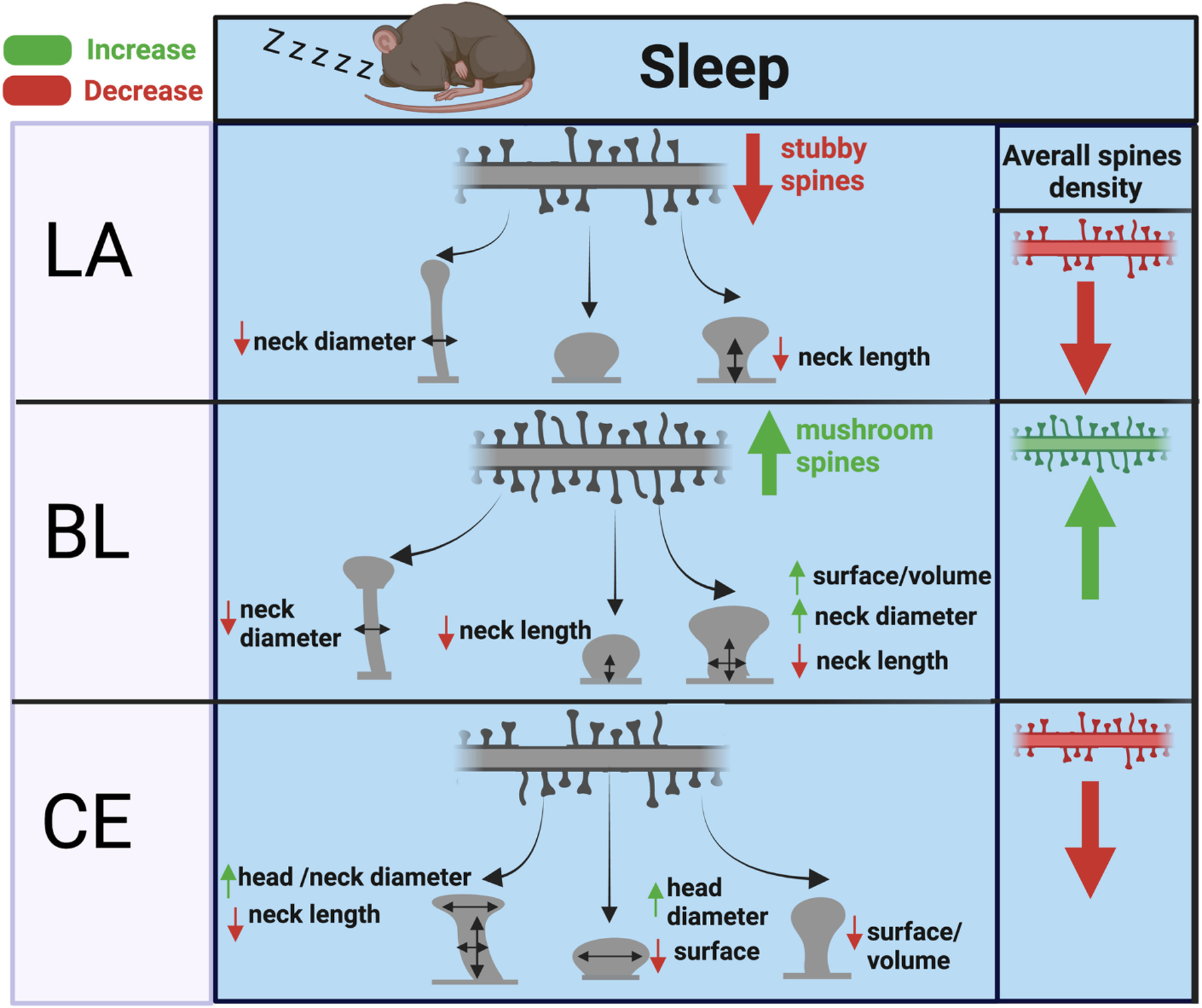
Summary of dendritic spine changes during sleep in the mouse amygdala. The diagram represents a summary of the working hypothesis of dendritic spine changes during sleep in the mouse amygdala based on our data from sleep deprived mice. Spine densities for each region are indicated by the branch with multiple spines in the upper part of each panel, and spine morphological subtypes are indicated by corresponding shapes (thin, subby, mushroom) in each panel. Mushroom, thin, and stubby spines in the BL are upscaled during sleep through increased spine density and corresponding decreases in neck diameter, neck length, and increased volume. In comparison, thin and stubby dendritic spines in the LA and CEA are largely downscaled during sleep through decreased spine density along with more selective changes in neck and head properties.

Our observed effects of sleep deprivation on dendritic spines in the BL are opposite to our previous observations in the hippocampus (Gisabella et al., 2020). Specifically, these results suggest that dendritic spines are broadly downscaled in the hippocampus during sleep but are instead strengthened in the basolateral amygdala. A closer examination points to a complex regulation of dendritic spines in both regions, however. In our previous study focused on sector CA1 of the hippocampus, the effects of sleep deprivation on hippocampal spines were region and branch specific (Gisabella et al., 2020). Studies from other groups also point to hippocampal subregion and branch specific changes, with evidence for upscaling of spines during sleep in the dentate gyrus and in the very distal apical branches of CA1 neurons (Raven et al., 2019; Bolsius et al., 2022). Similarly, our findings in the amygdala point to region and spine type specific changes, with evidence for upscaling of mushroom and stubby spines in the BL and downscaling of thin and stubby spines in the LA and CEA (Figure 6). These region-specific differences between the hippocampus and amygdala may reflect differential connectivity of these areas and their roles in sleep processes as well as memory replay during sleep. Stress is a factor that may contribute to differences between the amygdala and hippocampus. Chronic stress differentially effects on dendritic spines in these regions, with decreased spines in the hippocampus and increased spines in the basolateral amygdala (Vyas et al., 2002). This suggests that there are underlying molecular processes behind dendritic spine regulation between these areas that are involved in sleep as well as stress response.

In addition, sleep deprivation may serve as a stressor itself. Although our method of sleep deprivation mimicking gentle handling is designed to minimize stress, sleep deprivation itself is a stressor by nature. Thus, the effects of sleep deprivation may reflect, in part, the effect of stress on dendritic spines. However, our observed effects of sleep deprivation on dendritic spines in the amygdala (Figure 6) and hippocampus (Gisabella et al., 2020) overall are opposite to the reported effect of chronic stress on dendritic spines in these regions (Vyas et al., 2002). Similarly, our observed decreased density of GH immunoreactive cells in the LA of sleep deprived mice is in contrast to the increased GH expression reported in the amygdala following chronic stress (Meyer et al., 2014). Specifically, chronic stress was reported to increase dendritic spines in the amygdala, whereas our data shows that sleep deprivation results in downscaling of dendritic spines in this region. This suggests that sleep is involved in synaptic upscaling of mushroom spines in the basolateral amygdala. This synapse promoting effect of sleep in the amygdala may potentially associated with effects of slow wave sleep in this region, as this stage is more predominant in the first half of the inactive period whereas REM sleep is more prevalent in the latter half (Lacroix et al., 2018). Furthermore, SWS and sleep are involved in promoting memory consolidation processes (Stickgold et al., 2000; Miyamoto et al., 2017). Our findings consisting of spine type and region-specific upscaling or downscaling suggests that our results reflect complex effects of sleep that utilize overlapping molecular pathways which may also be utilized for dendritic spine regulation during stress.

### Relevance to psychiatric disorders

Several lines of evidence point to the involvement of synaptic alterations in the amygdala in memory consolidation and sleep disturbances in psychiatric disorders including PTSD and mood disorders (Ross et al., 1989; Leibenluft et al., 1996; Colombo et al., 1999; Bremner et al., 2000; Frodl et al., 2002; Drevets, 2003; Jackson et al., 2003; Lange and Irle, 2004; Harvey et al., 2005; Armitage, 2007; Benedetti et al., 2007; Raskind et al., 2007; Thompson et al., 2008; Karolewicz et al., 2009; Tottenham et al., 2010; Harb et al., 2012; McCarthy et al., 2012, 2013, 2016, 2019; Bunney and Bunney, 2013; Burton et al., 2013; Li et al., 2013; McClung, 2013; Palagini et al., 2013; Medina et al., 2014; Brownlow et al., 2015; Bunney et al., 2015; Pagani et al., 2016; Pantazopoulos et al., 2017, 2018; Nudell et al., 2019). Our data suggesting that mushroom spines in the amygdala are upscaled during sleep is in line with preclinical studies suggesting that sleep deprivation early on after a traumatic experience may alleviate the strength or consolidation of traumatic memories that contribute to development of PTSD (Wagner et al., 2006; Kuriyama et al., 2010; Cohen et al., 2012). In our previous studies, we demonstrated that chronic stress increases ghrelin-growth hormone signaling (Meyer et al., 2014) and in turn growth hormone expression increases dendritic spine density in the amygdala and predisposes amygdala neurons to encode fear memories (Gisabella et al., 2016), suggesting that stress may increase dendritic spines in this region in part through elevated growth hormone expression. In turn, chronic stress in rodents results in decreased dendritic spines in the hippocampus in comparison to increased spines in the amygdala, suggesting that growth hormone may be involved in this process (Vyas et al., 2002) and that the approach of sleep deprivation following a traumatic experience may alleviate the strength of the traumatic memory by disrupting the upscaling of dendritic spines in the BL that may be involved in consolidation of the traumatic memory. However, our data also suggests that sleep deprivation would increase dendritic spines in the LA and in the CEA, a key area involved in fear response output signaling. This suggests that sleep, in part, may contribute to resilience to stress by downscaling spines in these areas. Our evidence suggesting that mushroom spines in the BL are upscaled during sleep also suggests that sleep disturbances in schizophrenia may interfere with this process and contribute to synaptic alterations in this disorder. Several studies have reported decreases in sleep spindles together with memory consolidation deficits in patients with schizophrenia (Ferrarelli et al., 2007; Manoach et al., 2010, 2014). Furthermore, recent report points to altered diurnal molecular expression rhythms in the brain of subjects with SZ (Seney et al., 2019). In turn, deficits in the synaptic molecule synaptophysin, and in the synapse stabilizing structures perineuronal nets have been reported in the amygdala of subjects with schizophrenia (Pantazopoulos et al., 2010, 2015; Varea et al., 2012). Sleep and circadian rhythm disturbances in this disorder may thus contribute to impairment in emotional memory consolidation processes in this region differentially from the enhancement of emotional memories in PTSD.

## Limitations

The lack of dendritic branch and segment specific analysis in our study represents an important limitation. The complex organization of neurons and their dendritic branches in the amygdala compared to other regions made it challenging to follow branches from individual neurons and to establish sampling strategies. Neurons and their respective dendrites in other regions such as the hippocampus or cortex are organized in layers, resulting in reduced overlapping of dendritic processes that allows for more selective sampling. However, this was not possible in the amygdala. However, obtaining information regarding dendritic spines measures in this region in sleep-deprived animals using viral vector labeling in 3-dimensional quantification was important. Therefore, we analyzed all clearly visible viral vector labeled dendritic branches in our samples. Lack of cell type specificity for the dendritic spines sampled is another potential limiting factor in understanding how dendritic spines may be altered during sleep in specific populations of excitatory and inhibitory neurons in the amygdala. Our current study represents a broad overview of dendritic spine changes in sleep deprived animals in this region, similar to prior studies from several groups that examined spines in broad neuronal populations rather than selective neuronal subtypes (Maret et al., 2011; Raven et al., 2018, 2019; de Vivo et al., 2019; Spano et al., 2019; Gisabella et al., 2020). Future studies will focus on cell type specific and memory trace specific changes in amygdalar dendritic spines in sleep-deprived mice.

## Conclusion

Overall, our data represent the first study describing the effect of sleep deprivation on dendritic spines in the amygdala and provide insight into the morphological changes of dendritic spines during sleep in this region. Future studies into the molecular pathways underlying these region and spine type specific changes may provide insight into the consolidation of emotional memories during sleep and how this process may be affected in psychiatric disorders.

## Data Availability

The original contributions presented in the study are included in the article/supplementary material, further inquiries can be directed to the corresponding author.
